# Changing the routine: a move to patient initiated follow up to improve surgical outpatient clinic

**DOI:** 10.1111/ans.17676

**Published:** 2022-04-16

**Authors:** Joshua Balhorn, Bruce Su'a, James Jin, Sze‐Lin Peng, Maree Weston, Lincoln Israel, Andrew Connolly, Andrew G. Hill, Ashish Taneja

**Affiliations:** ^1^ The Department of General Surgery Middlemore Hospital Auckland New Zealand; ^2^ Faculty of Medical and Health Sciences The University of Auckland Auckland New Zealand

**Keywords:** colorectal surgery, follow‐up care, general surgery, haemorrhage, outpatient clinic

## Abstract

**Introduction:**

Patient initiated follow up (PIFU) allows patients to initiate a hospital follow up appointment on an ‘as required’ basis in contrast to the traditional physician‐initiated model. We present a clinical pathway for patients referred with rectal bleeding at a large tertiary public hospital in South Auckland, New Zealand and demonstrate the utility of PIFU and its impact on reducing follow up appointments.

**Method:**

The purpose of the pathway was to allow standardized care by the clinicians and allow for PIFU. Two separate protocols were developed ‐ ‘Painful PR bleeding’ and ‘Painless PR bleeding’. A new clinic (NC) was started following these protocols, and this was compared to historical controls (HC). The primary outcome was the rate of follow up appointments.

**Results:**

There were 133 patients in the NC and 135 in the HC, with significantly less follow ups in the NC (6% versus 45%, *p* < 0.0001). A small percentage of patients in the NC group were directly discharged (10%) whilst 70% of patients were discharged with a PIFU card. Thirty phone calls were made using PIFU, with 10 patients returning to clinic and 20 requiring advice and reassurance only. At 5 year follow up, there was a single colorectal malignancy found in both groups.

**Conclusion:**

Initiating a protocol that includes patient initiated follow up vastly reduces the need for routine return to clinic for the majority of patients, without sacrificing patient care. A protocolised approach to clinic for other areas in general surgery should be considered.

## Graphical abstract

Historically patients have often been followed up routinely for benign anorectal conditions, which places a large strain on outpatient clinic resources. Patient initiated follow up instead puts the onus on the patient to arrange further appointments if they need further input. Protocolising clinic visits allows for guidelines to follow so that every patient gets equal and quality care.

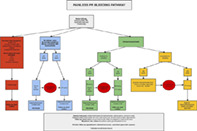

## Introduction

Increasingly public hospitals are faced with rising demands for healthcare services but limited healthcare resources. The drive to maintain the delivery of high quality services within such constraints has led clinicians and decision makers to look at tools that can help with planning, optimizing and reforming service processes. Outpatient clinics are an aspect of secondary healthcare that are a pressure point for increasing service demands and access times.

Clinical pathways are tools designed to help promote organized and efficient healthcare.^1^, ^2^, ^3^, ^4^, ^5^ They allow for implementation of evidence‐based guidelines, continuous quality improvement and standardization of processes as well as reduce clinical variation^1^ and equally importantly, reduce health inequities.^5^ One such process is the use of Patient Initiated Follow Up (PIFU). PIFU is an initiative that allows patients to initiate a hospital follow up appointment on an ‘as required’ basis in contrast to the traditional physician‐initiated model, thereby potentially reducing inappropriate regular follow up appointments. A systematic review, looking at the role of PIFU in the outpatient setting found that a reduction in the number of secondary healthcare outpatient appointments was achieved, whilst maintaining equivalent if not better patient satisfaction, quality of life and clinical outcomes across a range of chronic conditions without compromising patient care.^6^

In this study, we present a clinical pathway for patients referred to surgical outpatient clinics with rectal bleeding (PR bleeding) at a large tertiary public hospital in South Auckland, New Zealand and demonstrate the utility of PIFU and its impact on reducing follow up appointments.

## Methodology

### Important considerations

Increased demand for access to outpatient clinics for patients presenting with PR bleeding was the main driver for the development of the pathway. A key objective of reviewing patients with PR bleeding is to exclude the presence of a colorectal cancer. A study into patient perceptions of rectal bleeding by Kocher *et al*. revealed that approximately two‐thirds of patients had personal concerns about malignancy when they sought medical advice for rectal bleeding.^7^ Several studies have shown that other than PR bleeding or the presence of an abdominal mass, most bowel symptoms had low predictive values for the presence of colorectal cancer.^8^, ^9^, ^10^ A review of the 2003 NICE referral guidelines for patients suspicious for colorectal malignancy, in the UK by Eccersley et al.^11^ suggested that guidelines were not all encompassing and in fact most patients with identified colorectal cancer did not fit published referral criteria. Any pathway development must therefore take these facts into account.

### Pathway development

**Fig. 1:**
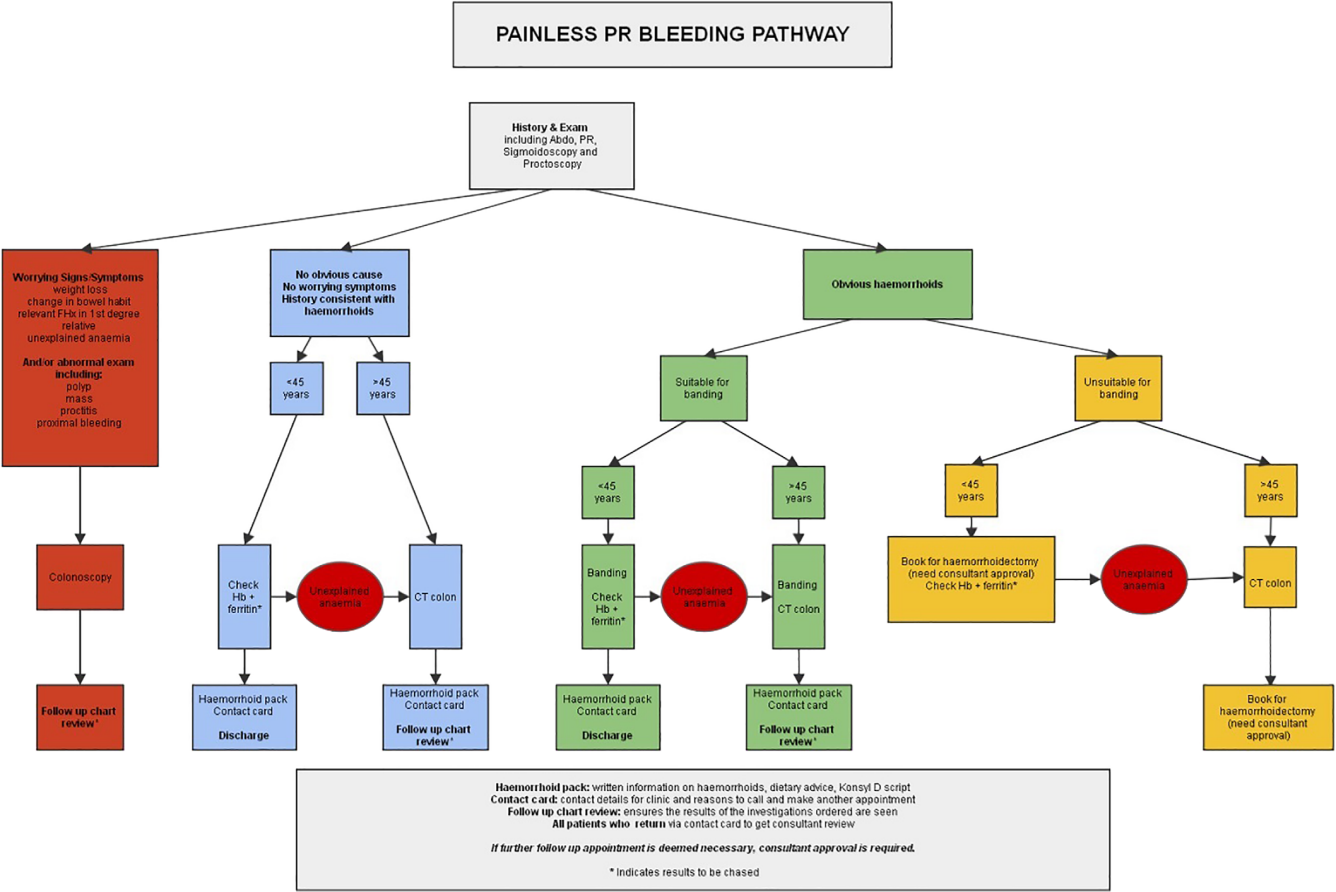
Painless PR bleeding pathway.

**Fig. 2:**
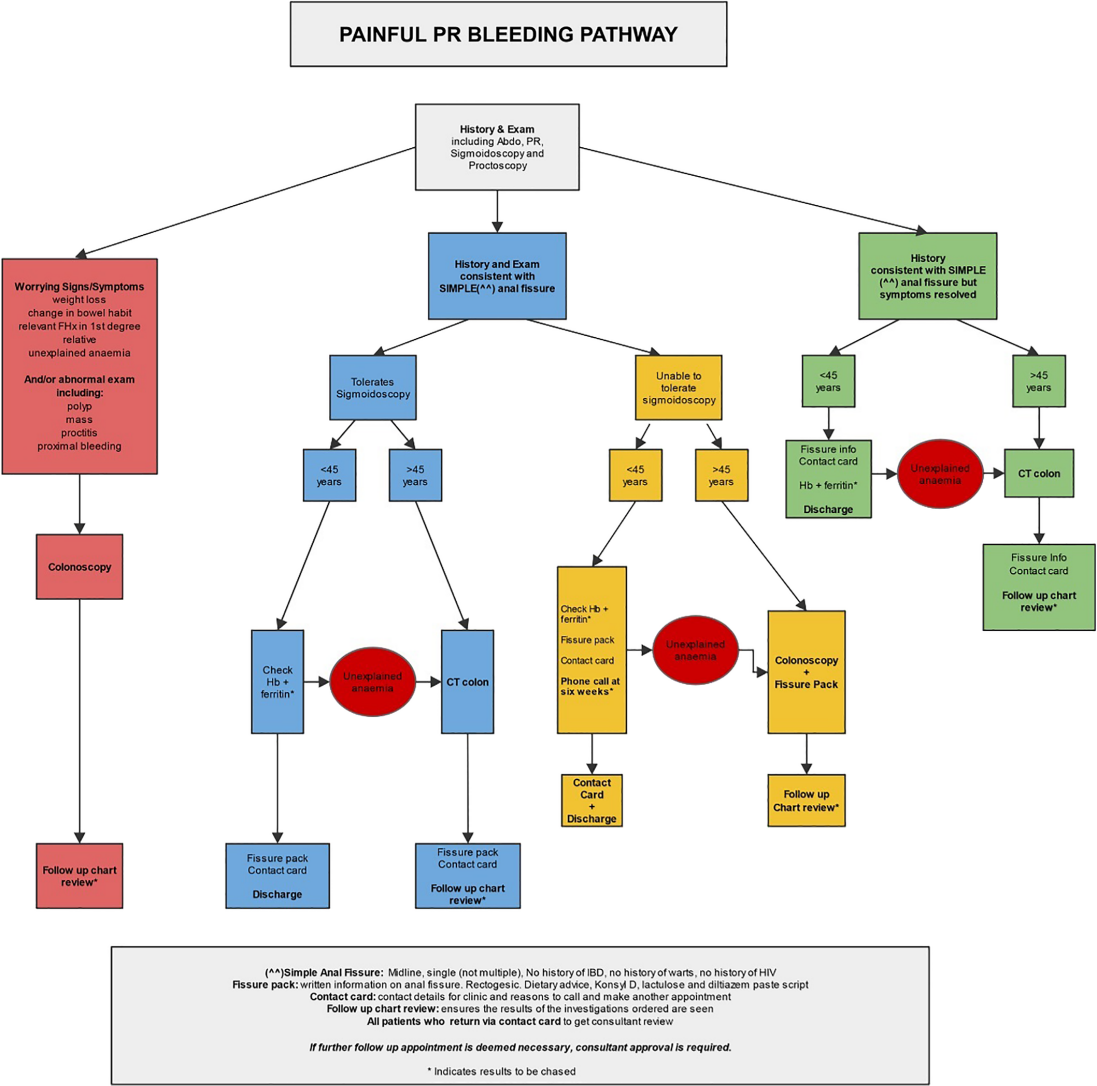
Painful PR bleeding pathway.

The purpose of the pathway was to allow standardized care by the clinicians and allow for PIFU. Two separate protocols were developed. One was for patients with ‘Painless PR bleeding’ (Fig. 1) and the other was for patients with ‘Painful PR bleeding’ (Fig. 2). The protocols were developed by the Colorectal Surgeons in the Department of General Surgery at Counties Manukau Health (CMH), taking into account local resource availability. It is important to note that access to colonoscopic investigation was restricted by high waiting times.

The key features of the new protocols included:

1. Patients with ‘high risk’ symptoms or signs were referred for colonoscopy. ‘High risk’ was defined by the presence of any one of an abdominal mass, unexplained anaemia, relevant family history in a first degree relative and altered bowel habit.
2. Any patient over the age of 45 without ‘high risk’ symptoms but with PR bleeding was referred for CT colonography (CTc).
3. All patients for whom investigations were requested were subjected to ‘chart review’ which was a review of the results of that particular investigation by the PR bleeding Nurse Specialist.
4. All patients were given a PIFU ‘card’ which they could use to contact the Nurse Specialist directly for any queries or to set up a follow up appointment as required. There was no time limit to the use of the PIFU card.

### Study design

Prior to the implementation of this study, patients with PR bleeding were seen in mixed clinics by both Colorectal Surgeons and General Surgeons. In order to test the practicality and utility of the new pathway, a once monthly dedicated clinic was set up to see approximately 14 new referral patients with PR bleeding only. This new clinic (NC) was an extra clinic, which ran in addition to the other clinics. All clinical consultations and examinations were supervised by two Colorectal Surgeons who participated in a rota of five surgeons. All patients were required to self‐administer a Microlax Enema™ prior to clinic arrival to help facilitate anorectal examination with rigid sigmoidoscopy and proctoscopy. If the preparation was noted to be poor, a repeat enema was administered by the nurse and the examination was repeated after half an hour.

The NC and the study began in November 2013. The study concluded in September 2014. Patients were non selectively allocated to the NC based on their position on the waiting list for outpatient clinic appointments. The remainder of the patients were seen in the usual clinics. Access to the newly developed protocols were available to all other surgeons in the department.

Only patients seen in the NC were recruited to the study. They were seen prior to their clinic appointment and consented for participation. Patients were excluded if they mistakenly presented to the NC as a follow up appointment or if they presented with colorectal symptoms other than PR bleeding (e.g., perianal fistula, rectal prolapse, pruritis ani *etc*.).

### Control group

The comparison group was a historical control of successive patients with PR bleeding, seen by the Colorectal Surgeons preceding December 2012. These patients were retrospectively identified through clinical records. The same exclusion criteria as above were applied.

## Outcomes

### Outcome measures

The primary outcome was the rate of follow up appointments. Secondary outcomes included rate of discharges, rate of elective operations and rate of investigations. Other relevant outcomes included the number of patients utilizing a PIFU appointment in the NC and the outcomes of the investigations that patients were referred for. Patients were subsequently followed up at 5 years from the conclusion of the study primarily to assess for development of an interval colorectal malignancy, and secondarily to review the rate of representation with PR bleeding.

### Sample size calculation

The rate of FU historically after an FSA was 45%. It was felt that a new protocol would help reduce the FU rate by a factor of at least 50%. Hence, assuming a power of 0.8 and a standard error rate of 0.05 the estimated sample size for the study group was calculated to be 68 patients.

### Ethics

Ethics approval was obtained from the University of Auckland Human Participants Ethics Committee (UAHPEC) and from the local institutional organization, Counties Manukau Health (CMH).

### Statistical analyses

All statistical analyses were performed using SPSS (IBM SPSS V22). Categorical variables were analysed using the Fisher's Exact test for between‐group comparisons. Continuous non‐parametric variables were analysed using the Mann Whitney U test for between‐group comparisons whilst for continuous parametric variables the student *t*‐test was used. Continuous parametric variables were presented as means with standard deviation, whilst continuous non‐parametric variables are presented as medians with inter‐quartile range. Analysis was performed on an intention to treat basis.

## Results

### Baseline data and primary diagnosis

Between November 2013 and September 2014, 11 NC were held. A total of 154 new patients presented through the NC. Of these, 14 patients were excluded from the study as their presenting complaint was not PR bleeding. Another seven patients did not provide consent for the use of their clinical information for the purposes of the study. A total of 133 patients were therefore ultimately used for prospective analysis in the NC arm of the study. In the historical control arm of the study, 135 consecutive patients with PR bleeding, seen by Colorectal surgeons preceding December 2012, were included. Table 1 shows the baseline characteristics and the diagnostic outcomes of the two groups. The mean ages were similar. A greater proportion of patients in the NC had haemorrhoids as their primary diagnosis, compared with the historical control (72% versus 59%, *p* = 0.001). The other diagnoses are of similar incidence. One case of rectal cancer was diagnosed in the NC on anorectal examination.

**Table 1:** Baseline characteristics and diagnoses of both arms

Characteristic	New clinic(*n* = 133)	Historical control(*n* = 135)	*p*‐value^†^
Age (mean)	48 ± 15	52 ± 15	0.001^‡^
Gender (%)	Male 63 (47%)	Male 52 (39%)	0.133
	Female 70 (53%)	Female 83 (61%)	
Diagnosis (%)			
1. Haemorrhoids	96 (72%)	79 (59%)	0.001
2. Fissure	22 (16%)	28 (21%)	0.001
3. Anal skin tag	7 (5%)	5 (4%)	
4. Rectal polyp	5 (4%)	7 (5%)	
5. Suspicious colon cancer	0 (0%)	4 (2%)	
6. Colorectal cancer	1 (1%)	1 (1%)	
7. Normal	0 (0%)	4 (3%)	
8. Other	2 (2%)	6 (4%)	

^†^ Fisher's exact test (2 sided).

^‡^ Independent samples *t*‐test.

### Outcomes of FSA

The primary outcome of the study was the rate of follow up appointments (Table 2). There were significantly less follow ups in the NC (6% versus 45%, *p* < 0.0001). A small percentage of patients in the NC group were directly discharged (10%) whilst 70% of patients were discharged with either a PIFU card or a PIFU card along with a chart review.

**Table 2:** Outcomes of FSA

Outcome of FSA	Specialised clinic (*n* = 133)	Historical control (*n* = 135)	*p*‐value†
Follow up appointment (%)	8 (6%)	61 (45%)	<0.0001
Discharged (%)	0 (0%)	54 (40%)	
Discharge with PIFU (%)	66 (49%)	0 (0%)	
Discharge with chart review and PIFU (%)	41 (30%)	0 (0%)	
Booked for surgery (%)	18 (14%)	21 (16%)	0.635

^†^ Fisher's exact test (2 sided).

### Treatment provided

There were also significant differences between the two arms with respect to the treatment provided (Table 3). Band ligation was performed more frequently in the NC group (36% versus 15%, *p* = 0.001). However, there were no significant differences in elective surgery rates (14% in NC versus 16% in HC).

**Table 3:** Treatment provided

Treatment	Specialised clinic (*n* = 133)	Historical control (*n* = 135)	*p*‐Value†
Banding (%)	48 (36%)	20 (15%)	0.001
Rectogesic (%)	12 (9%)	19 (14%)	0.066
Surgery (%)	18 (14%)	21 (16%)	0.635
Phenol (%)	0 (0%)	0 (0%)	
Laxative/diet advice/non‐specific advice only (%)	55 (41%)	74 (55%)	0.247

^†^ Fisher's exact test (2 sided).

### Investigations performed

In terms of investigations performed, Table 4 shows that the rates of colonic studies overall were similar between the two groups (45% in NC versus 40% in HC). There were significant more CT colonographies performed in the NC group (24% versus 5%, *p* = 0.0001).

**Table 4:** Investigations performed

Investigations	Specialised clinic (*n* = 133)	Historical control (*n* = 135)	*p*‐Value†
No investigation (%)	70 (53%)	74 (55%)	
Colonic examination (%)	60 (45%)	54 (40%)	
CTC	32 (24%)	7 (5%)	0.0001
Colonoscopy	28 (21%)	38 (28%)	0.345
Barium enema	1 (1%)	3 (2%)	
Recent colonoscopy	0 (0%)	6 (4%)	
Other^‡^	3 (2%)	7 (5%)	

^†^ Fisher's exact test (2 sided).

^‡^ Blood test only.

In the NC, colonoscopy was performed in 20% of cases overall. The most common finding was of benign colorectal polyps (41%). Normal colonoscopies or colonoscopies with only haemorrhoids were identified in 38% of cases. No malignancies were identified. The single patient in whom a rectal cancer was diagnosed in clinic had a rectal adenocarcinoma confirmed on colonoscopy.

CTc was used in 24% of patients in the NC (Table 5). Nineteen percent of patients who received a CTc went on to have a colonoscopy for polypectomy. A total of 31% were found to have extra colonic pathology but only 25% (eight patients) required further investigations. No extra‐colonic malignancies were identified.

**Table 5:** Outcomes of CTc

CTc outcome	Number (*n* = 32) (%)
Did not attend (DNA)	3 (9%)
Normal	19 (60%)
Haemorrhoids	0 (0%)
Diverticulosis	4 (13%)
Polyps (benign)	6 (19%)
Cancer	0 (0%)
Extra colonic findings	10 (31%)
Lung lesion	2
Pelvic lesion	3
Liver lesion	1
Renal pathology	2
Biliary pathology	2

### Utilization of PIFU

The PIFU card was utilized by 21 patients (16%) who a made a total of 30 phone calls to the nurse specialist (Table 6). In the majority of cases, phone advice and reassurance were all that was required. Two patients presented to the hospital emergency department with acute bleeding for which they were observed and did not require surgical intervention. A total of 10 follow up appointments were made for 6 patients.

**Table 6:** Utilization of PIFU

PIFU phone calls	Number
Total calls	30
Total patients using PIFU	21
Total presentations to Emergency department	2
Follow up appointments made	10
Reassurance and phone advice only	20

### 5 year follow up

There was one malignant neoplasm found in the NC group (1/127) at 5 year follow up. A CTc was performed following the NC and was normal; they underwent a colonoscopy for iron deficiency anaemia 3 years later which found a caecal tumour. This was treated with a right hemicolectomy. In the control group, a single malignancy was found (1/129) at the rectosigmoid junction 2 years following their index clinic appointment (where a colonoscopy had been performed prior). This was treated with a high anterior resection. Six patients in the NC were lost to 5 year follow up (three moved out of area, three died from unrelated conditions), and six in the HC (five patients died from unrelated conditions, one from a complication of their bowel surgery from cancer found at original clinic).

## Discussion

This study describes the development and utility of a clinical pathway to manage patients presenting to surgical outpatient clinics with PR bleeding and shows a significant reduction in overall follow up appointments through the utilization of PIFU.

There are only a handful of examples of ‘one‐stop’ dedicated rectal bleeding clinics in the published literature.^12^, ^13^, ^14^, ^15^ It is interesting and worth noting that most of the cited examples of one‐stop PR bleeding clinics have utilized flexible sigmoidoscopy as the main modality of investigation.^12^, ^13^, ^14^, ^16^ The UK Flexible Sigmoidoscopy Screening Trial (UKFSST) trial for colorectal cancer screening has shown the efficacy of a one‐off screening flexible sigmoidoscopic examination as a means of improving colorectal cancer detection and mortality.^17^

In the current study, the chief components of the two protocols for PR bleeding were the use of PIFU and the use of CTc as a one stop investigation for those patients with PR bleeding over the age of 45 and whose symptoms were not deemed as ‘high risk’. The role of CTc as a screening modality is evolving. Its sensitivity and specificity for detecting significant polyps (>10 mm) is very high according to a meta‐analysis by Sosna *et al*.^18^ Another meta‐analysis by Pickhardt *et al*.^19^ suggested a near 100% sensitivity rate for the detection of colorectal cancer. Furthermore, a large multicentre randomized study in the UK, the SIGGAR trial showed that CTc had equivalence in detecting large polyps or cancer when compared to the gold‐standard investigation, colonoscopy.^20^

Although access to CTc is superior compared to colonoscopy in the New Zealand health system and it has a very high sensitivity for larger polyps, it does have reduced sensitivity and specificity for small to diminutive polyps (<6 mm) and therefore its use for patients at ‘high risk’ for colorectal cancer is debatable.^20^, ^21^, ^22^ It is for this reason that patients who were clinically deemed as ‘high risk’ were referred directly for colonoscopy in both pathways. In this study, approximately 20% of patients required colonoscopy for the identification of polyps on CTc. In all cases the polyps identified were benign. At the same time nearly 25% of patients required further investigations for extra‐colonic incidental findings. These findings are consistent with ranges noted in other studies.^20^, ^23^, ^24^, ^25^, ^26^

The PIFU ‘card’ was given to all patients in the NC. Only 16% of patients utilized the PIFU card. In the vast majority of cases, a follow up appointment was not necessary and phone advice and reassurance was all that was required. One of the stated reasons for a high follow up rate in the historical control was to re‐check patient symptoms and provide reassurance to those who required it. It was also an opportunity to follow up on investigations that were requested, so that no abnormal results would be missed. The implemented pathway utilized the role of a Nurse Specialist to follow up on investigations requested by the surgical team and thereby also reducing the need for unnecessary routine follow ups.

One of the concerns with a lack of follow up is the risk of missing colorectal malignancy in patients who continue to be symptomatic. This risk is therefore partly mitigated by having a slightly lower age threshold for colonic investigation of 45 years of age, when compared to other international guidelines such as in Australia and USA.^27^, ^28^ It is also possible that some patients may not choose a follow up appointment, given the sensitive nature of the clinical examination in these clinics. It is worth noting however that the overall rates of colonic examination were similar between the two groups studied. A higher proportion of patients in the historical arm had colonoscopies compared to CTc. Given that there were no significant differences in the mean ages of both groups, it is likely that similar indications for colonic examination were applied in the historical control group. The implication of this finding is that the new pathway at the very least does not increase the risk of missing colorectal malignancy when compared to historical practice.

Longitudinal follow up at 5 years showed the rate of interval colorectal malignancy was essentially the same with a single cancer found in each group over this time period, supporting the notion above that malignant neoplasms will not be missed more often using the protocol. It is interesting to note that in both cases they had been investigated recently (within 3 years) with normal results; this highlights the need to consider repeat studies in all patients who continue to have symptoms despite their colon having been investigated within the last 5 years.

Despite several studies suggesting that other than PR bleeding or the presence of an abdominal mass, most bowel symptoms had low predictive values for the presence of colorectal cancer,^8^, ^9^, ^10^ only a single case of colorectal cancer was identified in the NC. The implication of this is that perhaps even rectal bleeding has a limited positive predictive value for the presence of colorectal malignancy.

This study has some limitations. We did not analyse the practice of the General Surgeons in the Department who also see outpatients with PR bleeding. In order to reduce the risk of selection bias, it was felt that study should compare the efficacy of the pathway between the same group of surgeons and hence the historical control group data was obtained for the same group of colorectal surgeons who participated in the rota for the NC.

## Conclusion

This clinical pathway for patients presenting to outpatient clinics for rectal bleeding demonstrates that standardized protocols that can help reduce variation in clinical practice. The use of PIFU is fundamental to this pathway and a significant reduction in follow up appointments was noted which has implications for extra capacity for outpatient clinics. A cost effectiveness analysis on the use of PIFU and the increased use of CTc would be useful in future investigations in this area.

## Conflicts of interest

The manuscript has not been published previously and is not under consideration elsewhere. All authors agreement with the content of the manuscript.

## Author contributions

**Bruce Su'a:** Data curation; project administration. **James Jin:** Data curation; project administration. **Sze‐Lin Peng:** Investigation; methodology. **Maree Weston:** Investigation; methodology. **Lincoln Israel:** Investigation; methodology. **Andrew Connolly:** Investigation; methodology. **Andrew G. Hill:** Conceptualization; supervision; visualization; writing – review and editing. **Ashish Taneja:** Conceptualization; data curation; formal analysis; writing – original draft; writing – review and editing.
